# Potential of Pine Bark to Replace Perlite in Coir-Based Substrates: Effects on Nutrient Uptake, Growth, and Phytochemicals in Lettuce Under Two Salinity Levels

**DOI:** 10.3390/plants14162577

**Published:** 2025-08-19

**Authors:** Gonçalo C. Dias, Rui M. A. Machado, Isabel Alves-Pereira, Rui A. Ferreira, Nazim S. Gruda

**Affiliations:** 1 MED—Mediterranean Institute for Agriculture, Environment and Development & CHANGE—Global Change and Sustainability Institute, Crop Science Department, School of Sciences and Technology, University of Évora, Pólo da Mitra, Ap. 94, 7006-554 Évora, Portugal; goncasdias123@hotmail.com; 2 MED—Mediterranean Institute for Agriculture, Environment and Development & CHANGE—Global Change and Sustainability Institute, Chemistry and Biochemistry Department, School of Sciences and Technology, University of Évora, Colégio Luís António Verney, Ap. 94, 7006-554 Évora, Portugal; raf@uevora.pt; 3 Division of Horticultural Sciences, University of Bonn, Auf dem Hügel 6, 53121 Bonn, Germany; ngruda@uni-bonn.de

**Keywords:** *Lactuca sativa* L., soilless cultivation, eco-friendly substrate, salinity, total phenols, ascorbate

## Abstract

Enhancing the sustainability of growing media is an important objective in soilless vegetable cultivation. Here, we evaluated the potential of pine bark to replace perlite in coir-based substrates for lettuce (*Lactuca sativa* L. cv. ‘Godzilla’) cultivation. The experiment followed a factorial design with two coir-based substrate blends—one amended with perlite and the other with pine bark—and two nutrient solution EC levels (1.5 ± 0.2 and 2.5 ± 0.2 dS m^−1^). The plants were cultivated in Styrofoam containers containing a substrate mix of 80% coir, 12% compost, and 8% perlite or pine bark (*v*/*v*). Replacing perlite with pine bark did not affect leaf macronutrient concentrations but increased leaf Fe and B levels. Increasing the EC of the nutrient solution increased leaf N, P, and K, with a significant rise in nitrogen. The substitution of perlite with pine bark in coir-based substrates did not affect leaf dry weight, head fresh weight, or chlorophyll content, total phenols, ascorbic acid, or proline, even under different salinity levels. The findings indicate the pine bark is an alternative to perlite, supporting comparable agronomic and quality outcomes in lettuce. Further research is recommended to confirm these results in crops with longer growing cycles.

## 1. Introduction

Sustainable production practices are increasingly crucial in soilless culture, particularly regarding the choice of substrate components [1,2,3]. Utilizing renewable, locally obtained resources can reduce both environmental harm and manufacturing expenses while promoting circular approaches within horticulture [4]. Consequently, carefully selecting and preparing substrates is crucial for achieving success in soilless cultivation, particularly in high-value crop sectors [5].

Perlite, a commonly used substrate component, is both energy-intensive to produce and relatively expensive [6]. Perlite is mined (mainly in countries such as the United States, Greece, and Turkey), thermally expanded, and shipped over long distances, resulting in considerable carbon emissions. The energy costs to produce and transport raise concerns about its environmental and economic sustainability. According to [7], milling alone requires approximately 6.70 kWh of electricity per metric ton, excluding the energy used in mining and transportation. Furthermore, spent perlite is difficult to recycle due to contamination with organic residues [8]. Pine bark can be an alternative, making it a more economical choice compared to the relatively expensive substrate, perlite [9]. It is a renewable byproduct of the forestry industry, widely available in countries like Portugal. A life cycle assessment made by Vinci and Mattia [10] has shown that bark-based substrates tend to have significantly lower environmental impacts than perlite. Overall, both materials are used to improve substrate aeration and drainage, but their physical and chemical properties differ significantly. Perlite is an inert, stable material with a pH typically ranging from 7.0 to 7.5. It does not provide mineral nutrients or buffering capacity, but it exhibits high capillarity [11]. Although its water-holding capacity depends on particle size, it is generally lower than that of most organic materials [12,13]. Pine bark, on the other hand, has a low cation exchange capacity [14], although it varies with particle size [15], and typically has a low pH. Pine bark has a negative charge due to the ionization of functional groups like carboxylic and phenolic [16]. Additionally, pine bark can degrade over time, leading to nitrogen immobilization [17,18]. When not composted or well-aged, it may contain phytotoxins [19]. These differences, alongside the characteristics of the nutrient solution, can affect water availability, pH, and electrical conductivity (EC), all of which are essential for nutrient uptake and plant performance [20].

Salinity in the root zone may reduce growth and enhance quality. Although generally applicable to leafy vegetables, exceptions such as lettuce and wild rocket may exhibit variations in consistency, which can potentially compromise certain quality traits [21]. In lettuce, eustress due to increased salinity in nutrient solution enhanced the accumulation of phenolic compounds and other secondary metabolites [22,23]. The objective of this study was to assess the agronomic and physiological responses of lettuce grown in coir-based substrates amended with either perlite or pine bark, under two nutrient solution salinity levels. Specifically, this study was to assess whether pine bark could serve as an alternative to perlite while maintaining nutrient absorption, growth, and quality attributes. We hypothesized that pine bark has the potential to replace perlite in coir-based substrates and would support comparable nutrient uptake, biomass production, and quality traits in lettuce. Additionally, we expected that this substitution would remain effective under increased salinity, indicating its potential as a sustainable component in soilless culture systems.

## 2. Results

### 2.1. Initial Growing Media Physicochemical Characteristics

The substitution of perlite by pine bark increased the electrical conductivity (EC), bulk density, and moisture content of the mix while decreasing its pH and total porosity (Table 1). The average EC values of the mixes with pine bark and perlite were 3.01 and 2.55 dS m^−1^, respectively (Table 1).

The pH of the mixes with pine bark (7.6) and perlite (7.5) was higher than the range considered suitable for cultivating vegetables (5.5 to 6.8) [24]. Pine bark increased the bulk density of the mix from 0.08 to 0.11 g/cm^3^ and decreased the total porosity from 91.41% to 89.66% (Table 1). Pine bark decreased the mass wetness of the mix, whereas the mix containing perlite exhibited a lower bulk density (0.08 g/cm^3^) and a higher moisture content.

### 2.2. Leachate EC and pH

**Figure 1:**
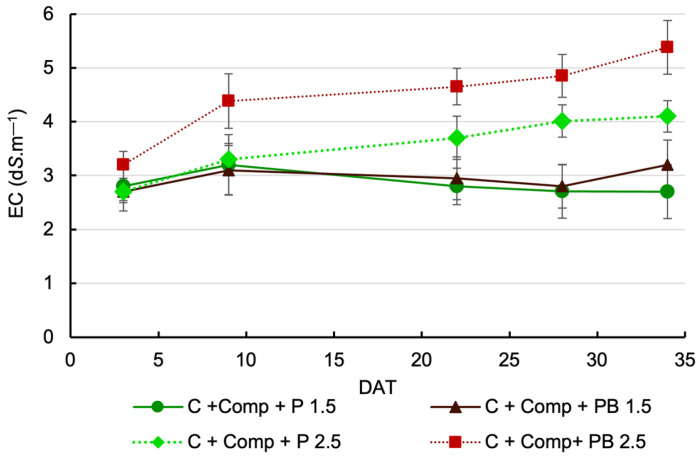
Effects of cultivation substrate mix and nutrient solution EC on average EC values of leachate above the initial electrical conductivity of the nutrient solution. Each symbol represents the mean of five replicates, and the error bars represent ±1 standard error. (C—coir, Comp—compost, P—perlite, PB—pine bark, DAT—days after transplantation).

The electrical conductivity of leachate above the initial EC of the nutrient solution was not affected by the interaction between treatments (*p* > 0.05) on any of the sampling dates (Figure 1). Replacing perlite with pine bark in the substrate mix had no significant effect on leachate EC when the nutrient solution had an EC of 1.5 dS m^−1^. In this treatment, leachate EC was slightly higher than the feed solution, ranging from 0.2 to 0.7 dS m^−1^ (Figure 1), which can be considered an acceptable level.

When the nutrient solution EC was 2.5 dS m^−1^, leachate EC remained consistently above the initial EC. EC values under this treatment were higher than those recorded under the 1.5 dS m^−1^ solution. In both substrate mixes, a clear upward trend in leachate EC was observed over time, indicating a loss of nutrients.

Except for the first sampling date, leachate EC was higher in the pine bark mix than in the perlite mix (Figure 1).

**Figure 2:**
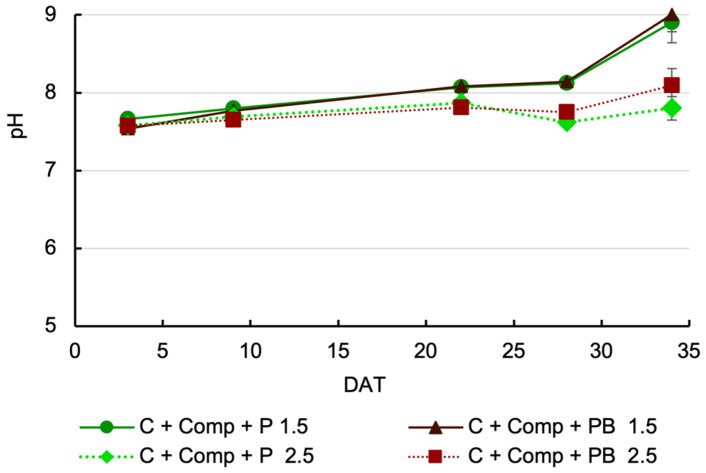
Effects of cultivation substrate mix and nutrient solution EC on pH of leachate. Each symbol represents the mean of five replicates, and the error bars represent ±1 standard error. (C—coir, Comp—compost, P—perlite, PB—pine bark, DAT—days after transplantation).

Leachate pH was not significantly affected by the interaction between treatments (*p* > 0.05) on any sampling date (Figure 2). Although pine bark is not inert like perlite, its inclusion in the mix did not significantly affect leachate pH. However, a significant decrease in pH (*p* < 0.001) was observed on the last two sampling dates, accompanied by an increase in EC in the nutrient solution. Across all treatments and dates, leachate pH values remained consistently higher than the pH of the incoming nutrient solution, which averaged 6.4 ± 0.5.

### 2.3. Growing Media pH and EC

**Figure 3:**
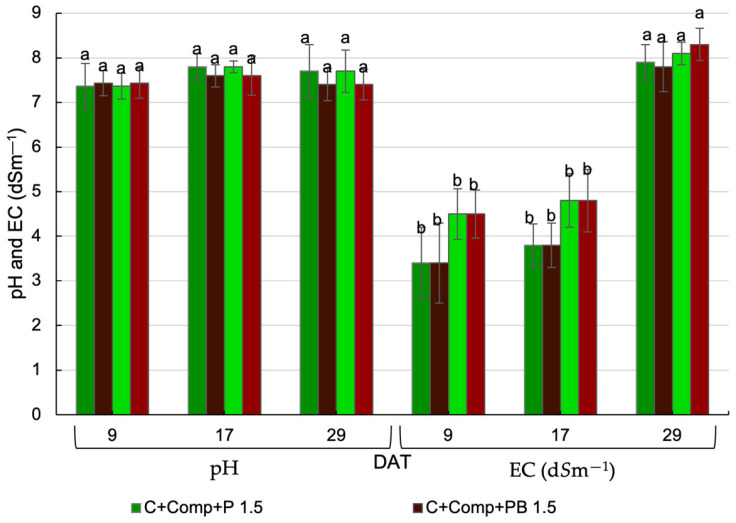
Effects of cultivation substrate mix and nutrient solution EC on pH and electrical conductivity of substrate solution (C—coir, Comp—compost, P—perlite, PB—pine bark, DAT—days after transplantation). Different letters for the same parameter indicate statistically significant differences (*p* < 0.05).

Substrate solution pH and EC collected at 9, 17, and 29 days after transplanting (DAT) were not significantly affected by the interaction between substrate mix and EC of the nutrient solution, indicating independent effects (Figure 3).

The substitution of perlite by pine bark in the substrate did not significantly influence the pH or EC of the substrate solution at any of the sampling dates (Figure 3). However, the nutrient solution EC had a significant effect on the EC of the substrate solution at 9 and 17 DAT. Specifically, higher EC values were observed in the treatment receiving the 2.5 dS m^−1^ nutrient solution compared to the 1.5 dS m^−1^ treatment (Table 2). On average, EC values measured across all three sampling dates were relatively high—particularly at 17 and 29 DAT—exceeding the recommended EC threshold for lettuce of 2.0 dS m^−1^ [25]. Despite these high EC average values, plants did not exhibit visible symptoms of salinity stress.

The pH and EC of the substrate, measured using the saturation extract method (1:5 *v*/*v*) after lettuce harvest, were not significantly affected by the interaction between treatments, mix composition, or the EC of the nutrient solution (Table 2). However, both pH and EC were significantly influenced by the sampling location (*p* < 0.001) (Table 2). pH and EC were higher on the lateral side perpendicular to the plant stem than on the center, near the stem (Table 2).

### 2.4. Leaf Nutrient Concentration

Leaf concentrations of N, P, K, Ca, and Mg were not influenced by the interaction of substrate and nutrient solution treatments (Table 3).

The replacement of perlite by pine bark in the substrate mix did not affect leaf macronutrient concentrations. In contrast, leaf N, K, and P content increased with the rise in the EC of the nutrient solution. The application of a nutrient solution with 2.5 dS m^−1^ increased leaf N content by 25% relative to plants grown with a nutrient solution of 1.5 dS m^−1^. The rise in leaf N content (0.9%) was greater than the increases in K (0.59%) and P (0.13%) (Table 3). Leaf Ca and Mg were not affected by the EC of the nutrient solution (*p* < 0.05) (Table 3).

Leaf micronutrient and Na content were not affected by the interaction of treatments (Table 3). Plants grown with pine bark had higher leaf Fe and B than those grown in perlite (Table 4). The nutrient solution EC only affected leaf B content, which increased with the rise in EC.

### 2.5. Photosynthetic Pigments

**Figure 4:**
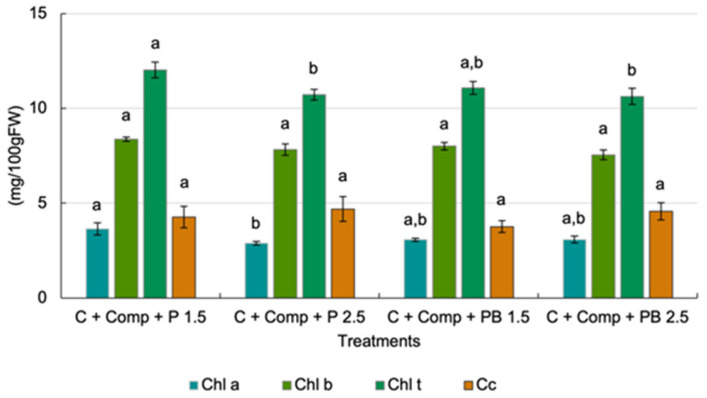
Effect of cultivation substrate mix and nutrient solution EC on photosynthetic pigments’ content. Chl a—chlorophyll a, Chl b—chlorophyll b, Chl t—total chlorophyll, Cc—carotenoids, C—coir, Comp—compost, P—perlite. PB—pine bark. Different letters for the same pigment indicate statistically significant differences (*p* < 0.05).

Leaf Chl a, Chl b, and Cc levels were unaffected by the interaction of treatments (Figure 4). This indicates that the effect of the EC of the nutrient solution on photosynthetic pigments has not been affected by the presence of perlite or pine bark in the mix. Chl a, b, and Cc content were not affected by the mix or the EC of the nutrient solution.

### 2.6. Plant Growth and Yield

Leaf dry weight, leaf number, foliar area, and fresh yield (kg m^−2^) were not affected by the interaction of treatments, nor by mix or nutrient solution EC (Table 4).

The nutrient solution EC and pine bark affected leaf nutrient content (Table 4); however, as plant growth was not affected, this may indicate that the leaf nutrient contents were within the sufficiency range for lettuce growth. The fresh yield of lettuce was high, ranging from 9.5 to 9.8 kg/m^2^ (Table 4).

### 2.7. Phytochemical Accumulation

Leaf phytochemical accumulation, including total phenolic content (TPC), ascorbate (AsA), glutathione (GSH), proline (Pro), water-soluble protein and proline dehydrogenase (PDH) (Table 5), was not significantly affected by the interaction between treatments, nor by the substrate mix or the nutrient solution EC (Table 5). The average TPC ranged from 22.24 to 28.66 mg GAE/100 g fresh weight (FW). The average AsA content ranged between 3.10 and 3.46 mg/100 g FW.

## 3. Discussion

The substitution of perlite with pine bark affected the EC values of the mix, but they remained within the range considered acceptable for soilless substrates [14,26]. In contrast, the pH values of both mixes exceed the optimal range for vegetable cultivation (5.5 to 6.8) [24], which may negatively affect lettuce plant nutrition. The increase in bulk density and decrease in total porosity with pine bark are consistent with its physical nature. However, both parameters remained within the acceptable range for substrate use, as total porosity values above 85% are considered ideal [26]. The lower mass wetness observed in the pine bark mix indicates reduced water retention capacity per unit of substrate weight, possibly due to pine bark’s coarser texture and lower intrinsic water-holding capacity compared to perlite [14]. Despite the pine bark affecting the mix properties, the changes were not substantial, indicating that a mix containing pine bark can be suitable for growing lettuce.

Replacing perlite with pine bark did not significantly alter leachate EC under low salinity conditions (1.5 dS m^−1^). In both substrate mixes, the leachate EC remained close to that of the nutrient solution, indicating that plants are properly taking up the nutrients. This implies that under moderate salinity, pine bark can function comparably to perlite in supporting nutrient dynamics within the root zone. However, under an EC of nutrient solution of 2.5 dS m^−1^, the pine bark mixes consistently exhibited higher leachate EC values compared to the mix with the perlite blend, especially in the last sampling dates. This is likely due to its higher initial EC (3.01 dS m^−1^) than perlite (2.55 dS m^−1^) (Table 1) and/or possibly greater retention of ions. Unlike perlite, which is inert, pine bark may contribute additional soluble salts or influence ion exchange processes within the substrate, thereby elevating EC in the leachate over time. Despite differences in leachate EC values, both mixes showed higher EC levels than the nutrient solution (2.5 dS m^−1^), indicating a loss of nutrients.

Leachate pH was not significantly affected using pine bark compared to perlite, suggesting that the organic nature of pine bark did not affect root-zone pH in the coir-based mix. This could be due to the relatively low proportion of pine bark in the mix (8% *v*/*v*). Both substrates increased leachate pH values, which were consistently higher than those of the incoming solution. According to [27], the pH of the drained solution can be either higher or lower than that of the nutrient solution. The uptake of nitrate can contribute to the release of OH^−^ ions, increasing the pH [28,29]. In this study, approximately 70% of the N in the nutrient solution was supplied as NO_3_^−^. The pH levels of the leachate—which may represent the pH of the solution within the growing medium in the two mixes at the different sampling dates—were higher than the optimal range for lettuce growth [30], which can negatively affect plant growth. A significant decline in leachate pH was observed at the final two sampling dates under high EC of nutrient solution (2.5 dS m^−1^). This may result from an increased release of protons from the soil exchange complex triggered by the higher concentration in the incoming nutrient solution.

Despite the average EC values of the substrate solution recorded on three sampling dates (Table 3) being too high, mainly at 17 and 29 DAT, exceeding the EC threshold for lettuce (2 dS m^−1^) [25], the plants did not exhibit visible symptoms of salinity stress. This may be related to the location of sample collection and/or the method of EC determination. The EC could be high because the substrate solution was collected some distance away from the emitter, at the periphery of the wetting bulb, where salt tends to accumulate. However, nutrient uptake occurs primarily at the center of the bulb, where moisture levels are higher and EC is lower [31], thereby reducing the salinity impact on the plants. Furthermore, the EC of the substrate solution collected with Rhizon samplers may be higher than that obtained using the saturated substrate extract method (SME), as Rhizon samplers extract the solution without additional dilution, preserving equilibrium conditions and maintaining higher concentrations of freely dissolved ions. The EC of the growing media was affected by the determination method [32,33]. Jeong et al. (2012) [33] reported that EC values from saturated media extract solutions (SME) were approximately 43% lower than those obtained by the Rizhon method. Further research is needed to clarify how sampling location and measurement method influence EC readings and their correlation with species-specific salinity thresholds.

The observed variation in pH and EC measured using the saturation extract method within the substrate profile can be attributed to salt accumulation patterns associated with the wetting front created during drip irrigation. When drip irrigation is used, salts tend to accumulate at the edges of the wet bulb, which likely explains the higher pH and EC values observed in the lateral. The placement of emitters near the plant stem may have helped to expand the wet bulb, thus preventing salt build-up around the stem. The placement of emitters near the plant’s stem prevents salt accumulation around the stem [34]. According to Ondrasek et al. [35], the location of emitters inside the pot can greatly influence the way moisture and salts are dispersed throughout the substrate. On the one hand, this study utilized two emitters, which may also contribute to the lack of difference in EC of the substrate. The use of more than one emitter contributes to the flushing of the salts [36] because the water and the salts extend more horizontally [37]. The configuration of the wet bulb formed by each emitter is determined by the physical properties of the substrate, including its texture and hydraulic conductivity, together with the emitter’s rate [38]. Despite the observed spatial differences in pH and EC, the lack of significant differences in mean values across treatments may contribute to the absence of treatment effects on lettuce yield and phytochemical accumulation.

The replacement of perlite with pine bark in a coir-based substrate does not affect leaf macronutrient content, supporting the feasibility of this substitution. In contrast, the N, P, and K content increased with nutrient solution EC, likely due to the higher availability resulting from the elevated nutrient concentration in the solution and/or the lower pH of the root medium (Figure 2).

Low pH can enhance the availability of P in the substrate [39]. Plants grown with pine bark had higher leaf Fe and B than those grown in perlite (Table 3). Fe and B concentrations in blueberry leaves also increased with the addition of Douglas fir bark to the media [40]. The absence of visual symptoms of nutrient disorders, combined with unaffected yield across treatments (Table 5), indicates that leaf nutrient levels remained within the sufficiency range for lettuce growth. The average values of leaf N, P, and K content determined in this study were above the lower sufficiency thresholds reported by [41] (3.3% for N, 0.35% for P, and 2.9% for K) at the pre-harvest stage.

Photosynthetic pigment concentrations were also unaffected by the treatments, indicating that the conditions in the pine bark mix were comparable to those in the perlite-based substrate. Leaf Chl b levels were higher than Chl a, which deviates from the typical pattern observed in most plant species. However, similar findings have been reported in lettuce by [42,43,44,45], which may be attributed to environmental conditions.

The absence of significant differences in plant growth and TPC, AsA, GSH, Pro, and PHD means that pine bark can replace coir-based substrates. The lack of response to nutrient solution salinity may be related to the fact that EC levels within the substrate were similar across treatments, possibly due to greater nutrient leaching when the EC of the nutrient solution was higher. This leaching could have been influenced by the type of container used, which allowed for lateral drainage. Katsoulas and Voogt [46] also noted that the effects of salinity strongly depend on the irrigation and drainage management methods applied.

The average TPC ranged between 22.24 and 28.66 mg GAE/100 g fresh weight (FW). These values fall within the ranges reported by Petropoulos et al. (18 to 203 mg GAE/100 g FW) and [47] (18.2–65 mg GAE/100 g FW) for green leaf lettuce. In contrast, ref. [42] observed higher average leaf total phenol concentrations in lettuce of the same cultivar, ranging from 54.52 to 138.96 mg GAE/100 g FW, in Batavia-type green leaf lettuce (Godzilla’) cultivated under similar cultural practices. This discrepancy could be attributed to differences in climatic conditions. Environmental factors such as temperature and radiation are known to influence the accumulation of phenolic compounds in vegetables [48]. The radiation and temperature are positively correlated with the content of phenolic acids and flavonoids in pigmented baby leaf lettuce, with levels increasing as the season progresses [49].

The novelty of this study lies in demonstrating that pine bark, a renewable and environmentally friendly by-product, has the potential to partially replace perlite in coir–compost substrates for lettuce cultivation. A key limitation, however, is that the experimental design evaluated only one substitution level (8% *v*/*v*), which restricts the assessment of dose–response effects and a full evaluation of its impact on lettuce yield and quality. Future studies should therefore test a wider range of pine bark proportions under different salinity levels, determine their effects on lettuce growth and quality, and assess whether this substitution remains suitable for crops with longer vegetative cycles.

## 4. Materials and Methods

### 4.1. Growth Conditions and Substrates

The experiment was carried out in a polycarbonate-covered greenhouse at the Herdade Experimental da Mitra (38°57′ N, 8°32′ W), University of Évora, Portugal. No supplemental lighting was used. Air temperature inside the greenhouse ranged from 8 to 27 °C, and daily solar radiation ranged from 34 to 248 W m^−2^·d^−1^.

The experiment comprised four different treatments: two coir-based mixes + compost with pine bark or perlite and two electrical conductivities (EC) of nutrient solution (1.5 ± 0.2 and 2.5 ± 0.2 dS m^−1^). Both mixes were composed of 80% coir pith (C) and 12% compost (Comp), expressed as volume per volume (*v*/*v*). One mix included 8% (*v*/*v*) of perlite (P), while the other included 8% (*v*/*v*) of pine bark (PB).

The following are the physical and chemical characteristics of the materials used, as provided by the manufacturer. The coir pith had a pH of 5.5 to 6.0, an EC greater than 1.5 dS m^−1^, granulometry 0–10 mm, total porosity = 95% *v*/*v*, air = 25% *v*/*v*, and cation exchange capacity (CEC) within the range of 60–120 meq/100 g. Compost (Nutrimais, Lipor Company, Baguim do Monte, Portugal) was prepared from horticultural materials, selected food waste from restaurants and cafeterias, forest harvest residues (branches and foliage), and green waste including flowers, grasses, and pruning debris. The EC and pH (1:5 compost: distilled water, w/v) were 5.4 dS m^−1^ and 9.0, respectively. According to the manufacturer, the compost used in this study is free of pathogens. Perlite (Knauf, Dortmund, Germany) has particles from 2 to 6 mm (coarse perlite), is pH-neutral, and is chemically inert. The pine bark (Siro, Mira, Portugal), obtained as a by-product of the country’s large maritime pine (*Pinus pinaster*), contained particles with a diameter of 8 to 15 mm and a pH (in CaCl_2_ solution) of 4.5. Pine bark had (expressed as a percentage of dry weight): organic matter (99.1%), C/N ratio (278), C (55.6%), N (0.20%), P_2_O_5_ (0.04%), K_2_O (0.11%), and Mg (0.05%).

**Figure 5:**
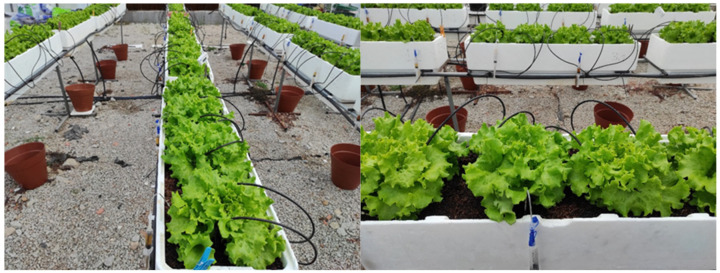
Appearance of the experimental setup and lettuce plants ten days before harvest.

On March 15, seedlings of lettuce (*Lactuca sativa* L. cv. Godzilla), type Batavia, 20 days after emergence, were planted into Styrofoam plant boxes along the central line, spaced 25 cm apart (16 plants/m^2^). The boxes (100 cm long × 25 cm wide × 10 cm high) were filled with 14 L of each mix at an approximate height of 6.5 cm. In this plant boxes drainage occurs laterally around the entire perimeter of the boxes. Treatments were arranged in a randomized complete block design with five replicate boxes per treatment (Figure 5).

The nutrient solution used contained 14 mmol L^−1^ NO_3_-N, 6.3 mmol L^−1^ NH_4_-N, 1.32 mmol L^−1^ P, 11 mmol L^−1^ K, 3.5 mmol L^−1^ Ca, 3.5 mmol L^−1^ Mg, 1.31 mmol L^−1^ S, 46 µmol L^−1^ B, 7.86 µmol L^−1^ Cu chelated by EDTA, 8.95 µmol L^−1^ Fe chelated by EDTA, 18.3 µmol L−^1^ Mn chelated by EDTA, 1 µmol L^−1^ Mo, 2 µmol L^−1^ Zn chelated by EDTA, 2.1 mmol L^−1^ Cl and 0.7 mmol L^−1^ Na. The pH of the nutrient solution was 6.4 ±0.5.

The two salinity levels were obtained by varying the concentration of the standard nutrient solution through the injection rate of the nutrient solution. From transplanting to 4 days after planting (DAP), a nutrient solution with an electrical conductivity of 1.1 dS m^−1^ was applied to all plants in the different treatments. After 4 days of planting, the nutrient solution was used with an electrical conductivity (EC) of 1.5 ± 0.2 and 2.5 ± 0.2 dS m^−1^ until one day before harvest. The irrigation schedule was optimized for the coir + compost + perlite mix. It was based on the substrate’s volumetric water content at the Styrofoam box control, measured using a soil moisture probe (SM105T, Delta Devices, Cambridge, UK), and the volume of water drained. The nutrient solution was applied three to eight times daily, with an average drainage (leaching fraction) of 10 to 25% for each application.

### 4.2. Measurements

Before planting, pH, electrical conductivity (EC), mass wetness, moisture content, total porosity, and bulk density of the mixes were measured. pH and EC were determined in the aqueous extract, which was prepared using a 1:5 substrate-to-water ratio (*w*/*v*). Moisture content, total porosity, and bulk density were measured using the porometer procedure outlined by [50], with four replications for each mix. Leachate from each planting box was collected five times during the crop cycle, and its pH and electrical conductivity (ECW) were measured using a potentiometer (pH Micro 2000, Crison Instruments, Barcelona, Spain) and a conductivity meter (LF 330 WTW, Weilheim, Germany).

The substrate solution was extracted using hydrophilic porous polymer Rhizon soil moisture samplers (Eijkelkamp Agrisearch Equipment, Giesbeek, The Netherlands), following the procedures described by [33,51], with adjustments made based on the specific conditions of this study. Three samplers were installed vertically in each Styrofoam box at a depth of 5 cm and positioned 8 cm away from the crop row, perpendicular to the plants. The samples were placed in substrate mixes and collected 24 h later. Soil solution was collected at 9, 17, and 29 days after transplanting (DAT), pooled by treatment, and analyzed for pH and electrical conductivity using a potentiometer (pH Micro 2000, Crison Instruments, Barcelona, Spain) and a conductivity meter (LF 330, WTW, Weilheim, Germany), respectively.

The harvest of lettuce heads took place on 20 April 2022, corresponding to 38 days after transplanting (DAT). The heads of the plants were cut off above the surface of the media. One head from each box was washed, oven-dried at 70 °C for 2–3 days, weighed, ground to pass through a 40-mesh sieve, and then analyzed for N, P, K, Ca, Mg, B, Fe, Mn, Zn, and Na. See [42] for a description of the procedures used for each analysis. Head samples were taken by slicing a 2 cm thick section at 6 cm above the base, ensuring that inner, middle, and outer leaves were included. The contents of photosynthetic pigments—chlorophyll a (Ch a), chlorophyll b (Ch b), and carotenoids (Cc)—were measured following the procedure outlined in Machado et al. [42].

After harvesting the lettuces, two substrate samples were collected from each box. One sample was taken near the stem, perpendicular to the lateral wall of the box, and the other 6 cm away from the perpendicular line of the plant stem. To collect the samples, a 3 cm square cut was made from the surface to the bottom of the box. The samples were homogenized, and pH and electrical conductivity were measured in an aqueous extract prepared at a 1:5 (*w*/*v*) ratio.

A 1 g portion of plant material from each of the four treatments and their replicates was weighed for the preparation of the methanolic extract (MW80). The material was ground in a mortar and homogenized in 8 mL of a methanol-water mixture (80:10, *v*/*v*) [52]. Extracts were clarified by centrifugation at 6500 *g* for 15 min at 4 °C. Aliquots were stored at −20 °C for later analysis of total phenolic compounds (TPC), ascorbate (AsA), and proline (Pro) content.

The aqueous extract in buffered solution (BE) was obtained by grinding 1 g of plant sample (from all four treatments and replicates) in a mortar with liquid nitrogen, followed by homogenization in 5 mL phosphate buffer (0.12 mM, pH 7.2). The homogenate was centrifuged at 10,000 *g* for 20 min at 4 °C. Aliquots of the supernatant were stored at −20 °C for later determination of protein and glutathione (GSH) content, as well as proline dehydrogenase (PDH) enzymatic activity [53,54].

Total phenolics in the MW80 extract were quantified following the procedure of Bouayed et al. [55]. For the reaction, the extract was combined with Folin–Ciocalteu reagent diluted 1:10 and a 7.5% sodium carbonate solution in a 1:5:4 ratio. After mixing with a vortex, the samples were left in darkness for 90 min before analysis.

Absorbance was measured at 760 nm. Phenolic content was calculated via graphical interpolation from a standard curve (GAE, 6 standards ranging from 0 to 50 mg L^−1^) and expressed as mg gallic acid equivalents (GAE) per 100 g fresh weight (FW).

Ascorbate quantification in the MW80 extract was performed as described by Cai and Tang [56]. A reaction mixture was prepared with MW80 extract, 5% TCA, absolute ethanol, 4% phosphoric acid in ethanol, 5% β-phenanthroline in ethanol, and 0.03% ferric chloride in ethanol (1:1:1:0.5:1:0.5). After vortexing, the mixture was incubated in a water bath at 30 °C for 90 min in the dark. Absorbance was measured at 534 nm. Ascorbate content was determined by interpolation from a calibration curve (AsA, 6 standards from 0 to 30 mg L^−1^) and expressed in mg AsA per 100 g FW.

Proline content was determined by molecular absorption spectrometry, as described by Gruda and Schnitzler [57] and Bates [58]. MW80 extract was reacted with glacial acetic acid and ninhydrin (1:1:1). After vortexing, the mixture was incubated in a 75 °C water bath for 1 h in the dark. Absorbance was measured at 546 nm. Proline content was interpolated from a calibration curve (L-proline, 6 standards from 0 to 20 mg L^−1^) and expressed in mg Pro per 100 g FW.

Glutathione levels were quantified using the protocol by Hissin and Hilf [59], which employed o-phthalaldehyde (OPT) to develop a measurable fluorophore. Diluted BE extracts or BE buffer (blank) were mixed with phosphate buffer (0.1 M, pH 8) containing 0.005 M EDTA and OPT. After vortexing, the mixture was left to stand for 10 min. Fluorescence was read at 25 °C (excitation: 350 nm; emission: 420 nm) using a Shimadzu RF-5001PC spectrofluorometer (Shimadzu Corpotation, Kyoto, Japan). Glutathione content was calculated from a standard curve (GSH, 6 standards from 0 to 100 mM) and expressed in mg GSH per g FW.

The catalytic activity of proline dehydrogenase (PDH, EC 1.5.5.2) was measured as described by Costilow [53]. A reaction mixture was prepared with sodium carbonate buffer (100 mM, pH 10.3), L-proline (2 mM), NAD^+^ (10 mM), and an appropriate amount of BE extract. NADH production was monitored by recording absorbance at 340 nm for 180 s at 30 °C using Double-beam spectrophotometer Hitachi U2001 with temperature control (Hitachi, Ltd., Tokyo, Japan). Enzymatic activity was calculated from the slope of the absorbance curve using the molar extinction coefficient of NADH (6.22 mM^−1^·cm^−1^) and expressed in nmol min^−1^ mg^−1^ protein.

The water-soluble protein content in the BE extract was calculated using the Lowry method [60]. A reaction mixture was prepared using 1.0 mL of Lowry reagent, 0.16 mL of 0.5 M NaOH, and 0.04 mL of BE extract. After vortexing and a 10 min rest period, 0.1 mL of diluted (1:2) Folin–Ciocalteu reagent was added. After vortexing again, the mixture was left to rest for 30 min. Protein content was determined from a calibration curve (bovine serum albumin, BSA, 6 standards from 0 to 200 mg mL^−1^).

All spectrophotometric measurements were performed using a Thermo Scientific Genesys 10S UV/V spectrophotometer (Thermo Fisher Scientific, Waltham, MA, USA) except for the measurement of PDH enzyme activity.

Data were analysed using analysis of variance in SPSS Statistics 29.0.1.0 (171) software (Chicago, IL, USA). Means were separated at the 5% level using Duncan’s new multiple range test.

## 5. Conclusions

This study shows that pine bark, an eco-friendly alternative, has the potential to replace perlite—a material with environmental and economic drawbacks—in coir-based substrates for lettuce cultivation, without affecting the yield and quality of lettuce. Replacing perlite with pine bark did not influence the effect of the nutrient solution electrical conductivity on substrate pH or the electrical conductivity of the substrate solution, which in combination with the nutrient solution, did not affect photosynthetic pigment content, leaf dry weight, fresh head yield, total phenolic compounds, ascorbic acid, or proline content in the leaves. The lack of response in electrical conductivity to the nutrient solution may be related to the irrigation methodology and the type of container used. Future research is advised to validate these results in crops with longer growing periods.

## Figures and Tables

**Table 1:** Initial mix physicochemical characteristics.

	Mix		
Parameter	C ^1^ + Comp + P	C + Comp + PB	Significance
pH	7.6 b ^2^	7.5 a	*
Electrical conductivity (d*S* m^−1^)	2.55 a	3.01 b	***
Bulk density (g cm^−3^)	0.08 b	0.11 a	***
Mass wetness (g water/g substrate)	5.22 a	4.60 b	***
Total porosity (%)	91.4 a	89.7 b	*
Moisture content (%, *w*/*w*)	79.56 b	81.06 a	*

^1^—C—coir, Comp—compost, P—perlite, PB—pine bark; ^2^—means followed by different letters within a column are significantly different. * and *** significant at *p* < 0.05 and 0.001 levels, respectively. Mean separation was performed using Duncan’s multiple-range test. Means are based on five replicates.

**Table 2:** Effect of sampling location on pH and EC of the cultivation substrate mixes at harvest time, measured near the plant stem (center) and on the lateral side of the container.

Sampling Location	pH	EC (d*S* m^−1^)
Center (near plant stem)	7.0 b ^1^	0.84 b
Lateral (perpendicular to the stem)	7.9 a	1.80 a
Significance	**	***

^1^—Means followed by different letters within a column are significantly different. ** and *** significant at *p* < 0.01 and 0.001 levels, respectively. Mean separation was performed using Duncan’s multiple-range test. Means are based on five replicates. EC = electrical conductivity.

**Table 3:** Effects of cultivation substrate mix and nutrient solution EC on leaf nutrient content.

Treatments	Leaf Macronutrients (%)					Leaf Micronutrients (μg·g^−1^)				
	N	P	K	Ca	Mg	Fe	B	Mn	Zn	Na ^3^
Mix (M)										
C+ Comp + P ^1^	4.0 b ^2^	0.75 b	6.29 b	0.85 a	0.29 a	90.9 b	34.6 b	55.7 a	67.3 a	0.36 a
C+ Comp + PB	4.1 b	0.67 b	6.33 b	0.81 a	0.28 a	106.6 a	43.1 a	52.5 a	65.2 a	0.38 a
EC (dS m^−1^)										
1.5	3.6 c	0.65 b	6.01 b	0.85 a	0.28 a	99.8 b	35.9 b	52.5 a	63.7 a	0.39 a
2.5	4.5 a	0.78 a	6.60 a	0.81 a	0.29 a	97.8 b	41.8 a	55.8 a	68.7 a	0.35 a
Significance										
M	NS	NS	NS	NS	NS	*	*	NS	NS	NS
EC	***	**	**	NS	NS	NS	*	NS	NS	NS
M x EC	NS	NS	*	NS	NS	NS	NS	NS	NS	NS

^1^—C—coir, Comp—compost, P—perlite. PB—pine bark; ^2^—means followed by different letters within a column are significantly different. *, **, and *** significant at *p* < 0.05, 0.01, and 0.001 levels, respectively, NS—not significant. Mean separation was performed using Duncan’s multiple-range test. ^3^—Even though sodium is not considered a micronutrient, it has been included here for convenience.

**Table 4:** Effects of cultivation substrate mix and nutrient solution EC on leaf dry weight, leaf number, area, and head fresh yield.

	Leaf Dry Weight	Leaves	Leaf Area	Head Fresh Yield
Treatments	(g/Plant)	(n°/Plant)	(cm^2^/Plant)	(kg·m^−2^)
Mix (M)				
C ^1^ + Comp + P	24.5 a ^2^	37.2 a	8854.9 a	9.5 a
C + Comp + PB	23.8 a	37.4 a	8486.9 a	9.8 a
EC (dS m^−1^)				
1.5	23.9 a	36.8 a	8806.4 a	9.7 a
2.5	24.0 a	37.8 a	8534.8 a	9.6 a
Significance				
M	NS	NS	NS	NS
EC	NS	NS	NS	NS
M x EC	NS	NS	NS	NS

^1^—C—coir, Comp—compost, P—perlite, PB—pine bark; ^2^—means followed by different letters within a column are significantly different. NS = not significant.

**Table 5:** Effects of cultivation substrate mix and nutrient solution EC on leaf total phenols, protein, AsA—ascorbate, GSH—glutathione, Pro—proline, PDH—proline dehydrogenase activity.

Treatments	TPC	WS-Protein ^3^	AsA	GSH	Proline	PDH
	(mg GAE/100 g FW)	(mg/100 g FW)				(nmol min^−1^/mg)
Mix (M)						
C + comp + P ^1^	27.69 a ^2^	195.9 a	3.46 a	1.22 a	1.51 a	29.40 a
C + comp + PB	22.48 a	224.1 a	3.10 a	1.15 a	1.44 a	32.30 a
EC (dS m^−1^)						
1.5	25.43 a	214.3 a	3.35 a	1.20 a	1.44 a	28.02 a
2.5	24.66 a	205.7 a	3.20 a	1.17 a	1.51 a	27.55 a
Significance						
M	^3^ NS	NS	NS	NS	NS	NS
EC	NS	NS	NS	NS	NS	NS
M x EC	NS	NS	NS	NS	NS	NS

^1^—C—coir, Comp—compost, P—perlite, PB—pine bark; ^2^—means followed by different letters within a column are significantly different. ^3^—WS—water-soluble; NS = not significant; Duncan’s multiple range test was used to distinguish between means (*p* < 0.05).

## Data Availability

The data supporting the findings of this study are contained in this article.
